# Durability of viral suppression with first-line antiretroviral therapy in patients with HIV in the UK: an observational cohort study

**DOI:** 10.1016/S2352-3018(17)30053-X

**Published:** 2017-05-04

**Authors:** Jemma O'Connor, Colette Smith, Fiona C Lampe, Margaret A Johnson, David R Chadwick, Mark Nelson, David Dunn, Alan Winston, Frank A Post, Caroline Sabin, Andrew N Phillips, UK Collaborative HIV Cohort (CHIC) Study, Jonathan Ainsworth, Sris Allan, Jane Anderson, Abdel Babiker, David Chadwick, Valerie Delpech, David Dunn, Martin Fisher, Brian Gazzard, Richard Gilson, Mark Gompels, Phillip Hay, Teresa Hill, Margaret Johnson, Sophie Jose, Stephen Kegg, Clifford Leen, Fabiola Martin, Mark Nelson, Chloe Orkin, Adrian Palfreeman, Andrew Phillips, Deenan Pillay, Frank Post, Jillian Pritchard, Caroline Sabin, Memory Sachikonye, Achim Schwenk, Anjum Tariq, Roy Trevelion, John Walsh, A Glabay, N Perry, S Tilbury, E Youssef, D Churchill, B Gazzard, M Nelson, R Everett, D Asboe, S Mandalia, F Post, H Korat, C Taylor, Z Gleisner, F Ibrahim, L Campbell, R Gilson, N Brima, I Williams, M Johnson, M Youle, F Lampe, C Smith, R Tsintas, C Chaloner, S Hutchinson, C Sabin, A Phillips, T Hill, S Jose, A Thornton, S Huntington, J Walsh, N Mackie, A Winston, J Weber, F Ramzan, M Carder, C Orkin, J Lynch, J Hand, C de Souza, J Anderson, S Munshi, J Ainsworth, A Schwenk, S Miller, C Wood, C Leen, A Wilson, S Morris, M Gompels, S Allan, A Palfreeman, K Memon, A Lewszuk, D Chadwick, E Cope, J Gibson, S Kegg, P Main, S Mitchell, M Hunter, M Dhillon, F Martin, S Russell-Sharpe, S Allan, A Harte, S Clay, A Tariq, H Spencer, R Jones, J Pritchard, S Cumming, C Atkinson, V Delpech

**Affiliations:** a Research Department of Infection and Population Health, UCL, London, UK; b Royal Free London NHS Foundation Trust, London, UK; c Centre for Clinical Infection, James Cook University Hospital, Middlesbrough, UK; d Department of Sexual Health and HIV, Chelsea and Westminster Hospital, London, UK; e Department of Medicine, Imperial College and St Mary's Hospital, London, UK; f Department of Sexual Health and HIV, King's College Hospital NHS Foundation Trust, London, UK

## Abstract

**Background:**

The length of time that people with HIV on antiretroviral therapy (ART) with viral load suppression will be able to continue before developing viral rebound is unknown. We aimed to investigate the rate of first viral rebound in people that have achieved initial suppression with ART, to determine factors associated with viral rebound, and to use these estimates to predict long-term durability of viral suppression.

**Methods:**

The UK Collaborative HIV Cohort (UK CHIC) Study is an ongoing multicentre cohort study that brings together in a standardised format data on people with HIV attending clinics around the UK. We included participants who started ART with three or more drugs and who had achieved viral suppression (≤50 copies per mL) by 9 months after the start of ART (baseline). Viral rebound was defined as the first single viral load of more than 200 copies per mL or treatment interruption (for ≥1 month). We investigated factors associated with viral rebound with Poisson regression. These results were used to calculate the rate of viral rebound according to several key factors, including age, calendar year at start of ART, and time since baseline.

**Results:**

Of the 16 101 people included, 4519 had a first viral rebound over 58 038 person-years (7·8 per 100 person-years, 95% CI 7·6–8·0). Of the 4519 viral rebounds, 3105 (69%) were defined by measurement of a single viral load of more than 200 copies per mL, and 1414 (31%) by a documented treatment interruption. The rate of first viral rebound declined substantially over time until 7 years from baseline. The other factors associated with viral rebound were current age at follow-up and calendar year at ART initiation (p<0·0001) and HIV risk group (p<0·0001); higher pre-ART CD4 count (p=0·0008) and pre-ART viral load (p=0·0003) were associated with viral rebound in the multivariate analysis only. For 1322 (29%) of the 3105 people with observed viral rebound, the next viral load value after rebound was 50 copies per mL or less with no regimen change. For HIV-positive men who have sex with men, our estimates suggest that the probability of first viral rebound reaches a plateau of 1·4% per year after 45 years of age, and 1·0% when accounting for the fact that 29% of viral rebounds are temporary elevations.

**Interpretation:**

A substantial proportion of people on ART will not have viral rebound over their lifetime, which has implications for people with HIV and the planning of future drug development.

**Funding:**

UK Medical Research Council.

## Introduction

The goal of antiretroviral therapy (ART) for people infected with HIV is to achieve and maintain continuous maximal virological suppression to allow immune reconstitution, minimise the risk of resistance emergence,^1^, ^2^, ^3^, ^4^ prevent HIV-related morbidity and mortality, and to prevent transmission.^1^, ^2^, ^3^, ^4^, ^5^, ^6^, ^7^, ^8^, ^9^ Most people starting treatment now achieve virological suppression within around 3–6 months.^10^, ^11^, ^12^ Rates of viral rebound have decreased over the years and the risk of rebound decreases with increasing duration of viral suppression.^13^, ^14^, ^15^, ^16^, ^17^ However, although current regimens seem to be durably effective, the extent to which people living with HIV will be able to sustain sufficient adherence to maintain viral suppression on ART in the long term is unknown. Few studies have estimated the long-term rate of viral rebound in people who initially achieved viral suppression. No study that we could identify has investigated the factors associated with the rate of viral rebound and used such findings to project rates of viral rebound over a lifetime or calculated the implied probability of requiring only one ART regimen in a lifetime. With the number of people on ART worldwide approaching 20 million, this information is of public health significance worldwide.

The UK Collaborative HIV Cohort (UK CHIC) Study includes data from long-term follow-up of a large and diverse cohort of people living with HIV. In the analysis reported here, we aimed to estimate the long-term rate of first viral rebound and to identify associated factors in people who achieve viral suppression after ART initiation. We then used these rate estimates to predict long-term durability of viral suppression.

**Research in context**

**Evidence before this study**

Many studies have looked at viral load outcomes in people on antiretroviral therapy (ART) and they have generally described high viral load suppression with modern regimens. We searched Web of Knowledge using the following search terms: “hiv*” AND “vir*” AND (“rebound” OR “failure”) AND “rate” AND “antiretroviral”, restricting to English language with no publication date restrictions, on Sept 28, 2016. Very few studies have estimated the long-term incidence of viral rebound in people who have initially achieved viral suppression. No studies that we could identify have assessed the factors associated with viral rebound and used such findings to project incidence of viral rebound over a lifetime or calculated the implied probability of requiring only one ART regimen in a lifetime.

**Added value of this study**

We show that in individuals who start ART and achieve viral load suppression on a first-line regimen, incidence of first viral rebound is low and declines over at least 7 years before stabilising at a low level—as low as 1% per year in some demographic groups.

**Implications of all the available evidence**

A substantial proportion of people on ART will not have viral rebound on their first-line regimen over their lifetime. Sufficient adherence to achieve lIfetime suppression of HIV viral replication is achievable with modern antiretroviral regimens, further supporting the strategy of maximising diagnosis of HIV and ART initiation, for personal and public health benefit.

## Methods

### Study design and participants

UK CHIC is an ongoing multicentre cohort study that collates routinely collected data from HIV-positive individuals aged 16 years and older who have been seen for care at at least one of 21 clinical centres in the UK. The study details are described elsewhere.^18^ In brief, centres collect data on demographic information, ART treatment history, laboratory results, and AIDS diagnoses. Start and stop dates for ART drugs are obtained from a review of clinical notes in some clinics and from pharmacy records in others.

These analyses are based on the 2014 update of the UK CHIC Study. An individual was eligible for inclusion if they met the following criteria: started ART for the first time (with a regimen containing three or more antiretroviral drugs) between Jan 1, 1998, and May 31, 2013; had at least 9 months of follow-up since the start of ART; and was on ART 9 months after ART initiation with a most recent viral load (measured 3–9 months after starting ART) of 50 copies per mL or less. We excluded anyone with pre-ART viral loads (most recently measured viral load at ART initiation) of 50 copies per mL or less (or if undetectable when the lower limit of detection was >50 copies per mL) because we suspected unrecorded previous ART use. The baseline for these analyses was defined as 9 months after ART initiation.

The UK CHIC Study received approval by the West Midlands multicentre research ethics committee (MREC/00/7/47) and by local ethics committees and, following UK regulations, does not require individual informed consent.

### Procedures

Viral rebound was defined as either a single viral load of more than 200 copies per mL or a documented treatment interruption (a record of no ART use for ≥1 month). We used a single viral load to define viral rebound because practical data limitations can mean that a confirmatory viral load is not available within a suitable timeframe and because physician intervention might occur after the first viral load of more than 200 copies per mL. However, a single viral load value of more than 200 copies per mL can often be followed by a value of 50 copies per mL or less without any change in regimen, so we also calculated the proportion of people who had viral rebound who went on to achieve resuppression without a change in ART regimen.

Individuals were followed up from baseline until whichever of the following events occurred first: viral rebound; start of a gap in viral load measures of at least 12 months; last follow-up (date of last viral load measurement or last update on treatment information); or 15 years from baseline.

### Statistical analysis

We calculated the incidence of first viral rebound (number of first viral rebounds per person-year of follow-up) stratified by time since study baseline.

We did several sensitivity analyses in which we considered alternative definitions of first viral rebound with the following modifications: not counting treatment interruption as a rebound; censoring follow-up at the date of treatment interruption instead of defining it as viral rebound; including loss to follow-up as a component of the viral rebound endpoint (ie, a gap in viral load measurements of ≥12 months or follow-up no longer occurring at a UK CHIC site, which cover approximately 40% of people diagnosed with HIV after Jan 1, 2013, in the UK); defining viral rebound as the first of two consecutive viral load measurements of more than 200 copies per mL, including treatment interruption; and without censoring follow-up when a gap in viral load measurements of more than 12 months occurred.

Poisson regression (PROC GENMOD in SAS version 9.4) was used to identify factors potentially associated with viral rebound. Follow-up was divided into 1 month periods for this analysis. Factors considered were age at a given month of follow-up (which we termed current age), HIV-acquisition risk group, time from baseline to a given month of follow-up, calendar year at ART initiation, and pre-ART viral load and CD4 count. We categorised the length of time from baseline to a given month of follow-up into groups (<1 year, 1–2 years, 2–3 years, 3–4 years, 4–5 years, 5–6 years, 6–7 years, and >7 years). Times from baseline to follow-up longer than 7 years were combined into a single category, because our analyses revealed no statistically significant trend over follow-up times after 7 years. We recognise that the absence of statistically significant evidence for a decline in viral rebound rate after 7 years does not exclude the possibility that there was some decline in rate that was not detected because of low statistical power. However, for our projections we adopted the conservative assumption of no further decline. We used a similar approach to defining an upper cutoff for both calendar year at start of ART (2008) and current age at follow-up (45 years). Calendar year at start of ART was categorised into Jan 1, 1998, to Dec 31, 1999; 2000–01; 2002–03; 2004–05; 2006–07; and Jan 1, 2008 onwards. Current age was categorised into younger than 20 years; 20–25 years; 25–30 years; 30–35 years; 35–40 years; 40–45 years; and 45 years and older. For both calendar year and age, lower rates of viral rebound were associated with later calendar year and older age, respectively, and so again our approach was conservative in assuming a constant rate above the highest cutoff. In the first multivariable model we included the starting regimen and the pre-ART viral load and CD4 count. In the second multivariable model we excluded these factors to give a simpler model with no missing data, to be used for projecting future viral rebound rates for individuals with different characteristics. Projections for these individuals were generated by use of model parameter estimates to obtain an estimated incidence of first viral rebound for each year of a person's life, taking account of their age at assessment and the calendar year at start of ART, and the time since baseline. These estimates were then used to determine the cumulative probability of not having viral rebound, which enabled calculation of estimates of cumulative survival without viral rebound and the inverse, cumulative risk of having viral rebound, by a given age. These estimates are conditional on the person living to that age. We calculated the risk of viral rebound and rebound-free survival rather than estimating the probability of having survived to and avoided viral rebound by a particular age because the latter is dependent on the mortality rate. Estimates of the probability of having survived to and avoided viral rebound by a specific age can be derived by readers from our estimated rates of viral rebound for any given set of age-specific mortality rates.

All analyses were done with SAS software (version 9.4; SAS Institute, Cary, NC, USA). All tests of significance were two-sided.

### Role of the funding source

The funder of the study had no role in study design, data collection, data analysis, data interpretation, or writing of the report. The corresponding author had full access to all the data in the study and had final responsibility for the decision to submit for publication.

## Results

We identified 27 734 people who started ART with three or more drugs on or after Jan 1, 1998. Of these, 19 094 had at least one viral load measurement between 3 months after the start of ART and baseline (9 months after start of ART), and at least 1 day of follow-up after baseline; of these 19 094 individuals, 16 101 (84%) also had a viral load of 50 copies per mL or less at baseline, and were included in the analysis (table 1). The earliest baseline date was Oct 1, 1998, and the latest was Feb 21, 2014. The latest end of follow-up date was Nov 22, 2014. The median number of viral load measurements per person was seven (IQR 3–17), and the median number of years of follow-up was 2·6 (1·1–5·2), with 13 463 person-years after year 5 and 1847 person-years after year 10. Viral load monitoring occurred at a median frequency of every 4·4 months (IQR 3·2–5·0). The 16 101 people included in the analysis differed from the 11 633 excluded people in the proportion of women (4050 [25%] in the participants *vs* 3813 [33%] in the excluded individuals) and the proportion of men who have sex with men (MSM; 8642 [54%] *vs* 5107 [44%]); the two groups did, however, have a similar median age (37 years [IQR 32–44] *vs* 35 years [30–42]).

**Table 1:** Characteristics of people included in the analyses

		**Participants (n=16 101)**
Age at start of ART (years)		37 (32–44)
Female sex		4050 (25%)
HIV risk-group, ethnicity, sex		
	Black African heterosexual men	1529 (9%)
	Black African heterosexual women	2687 (17%)
	Men who have sex with men	8642 (54%)
	Non-black heterosexual men and women	2144 (13%)
	Injectable drug users (past and present)	284 (2%)
	Unknown	815 (5%)
Women pregnant at start of ART		372 (2%)
Starting regimen type		
	Non-nucleoside reverse transcriptase inhibitor based	11 599 (72%)
	Ritonavir-boosted protease inhibitor based	3333 (21%)
	Unboosted protease inhibitor	654 (4%)
	Nucleoside or nucleotide-only	515 (3%)
Calendar year of start of ART		
	1998–99	1058 (7%)
	2000–01	1358 (8%)
	2002–03	1816 (11%)
	2004–05	2183 (14%)
	2006–07	2439 (15%)
	2008–09	3111 (19%)
	2010–11	2866 (18%)
	2012–13	1270 (8%)
Pre-ART viral load (copies per mL)*		
	51–500	464 (3%)
	501–5000	1069 (7%)
	5001–20 000	2063 (14%)
	20 001–100 000	4852 (33%)
	100 001–500 000	4864 (34%)
	>500 000	1174 (8%)
Pre-ART HIV viral load (log_10_ copies per mL)*		4·9 (4·3–5·3)
Pre-ART CD4 count (cells per μL)†		217 (120–310)
Time from baseline‡ to end of follow-up (years)		2·6 (1·1–5·2)

Data are median (IQR) or n (%). ART=antiretroviral therapy.

* Available for 14 486 individuals.

† Available for 14 519 individuals.

‡ Baseline is defined as 9 months after ART inititiation.

4519 first viral rebounds were observed over 58 038 person-years, giving an overall rate of first viral rebound of 7·8 (95% CI 7·6–8·0) per 100 person-years. Of the 4519 participants with a first viral rebound, 3105 (69%) had a single viral load of more than 200 copies per mL and 1414 (31%) had a documented treatment interruption. Of the 3105 people who had a viral load of more than 200 copies per mL, 2999 (97%) had a subsequent viral load value available. 1377 (46%) of the 2999 people achieved virological resuppression, and 1322 (44% of the 2999 with a subsequent viral load; 29% of all 3105 with viral rebound) achieved virological resuppression without a change in ART regimen. Of the 1622 people who did not have virological resuppression at the subsequent viral load measure, the median value of the subsequent viral load was 906 copies per mL (IQR 209–13 461); 792 individuals (49%) had more than 1000 copies per mL, 436 (27%) had more than 10 000 copies per mL, and 157 (10%) had more than 100 000 copies per mL.

Incidence of viral rebound clearly decreased over time since baseline (figure). The rate was 12·6 per 100 person-years in year 1 and declined to 2·5 per 100 person-years in years 10–15 combined. To identify the timepoint at which the decline in viral rebound rate was no longer statistically significant, we first fitted a univariable Poisson regression model of the association between time from baseline and the rate of viral rebound. Overall, considering all follow-up time from baseline to 15 years after, a highly statistically significant declining trend in rate was seen (16% per year; p<0·0001). When we restricted inclusion of follow-up to person-years after year 1 from baseline, the declining trend remained (13% per year; p<0·0001). When we restricted inclusion to follow-up after 2 years (11%; p<0·0001), 3 years (11%; p<0·0001), 4 years (11%; p<0·0001), 5 years (11%; p<0·0001), or 6 years (9%; p=0·003) from baseline, the statistically significant decline persisted. However, when we restricted inclusion to follow-up after 7 years (5% decline; p=0·27) or 8 years (3% decline; p=0·68), the trend was no longer statistically significant. By restricting inclusion to follow-up before year 7, the average decline in rate of rebound between year 1 and year 7 was 18% per year (unadjusted rate ratio 0·82 per year, 95% CI 0·80–0·84; p<0·0001). The strong effect of time from baseline persisted, and even strengthened, after adjustment for other covariates (table 2).

**Figure:**
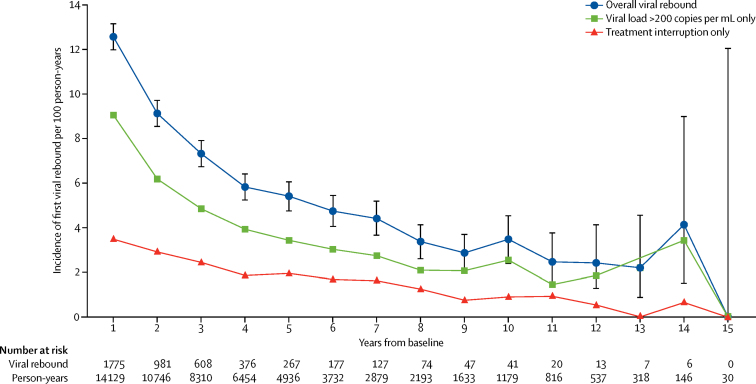
Incidence of first viral rebound by time since study baseline (9 months after start of ART) Error bars show 95% CI.

**Table 2:** Factors associated with first viral rebound

		**Univariable**	**Multivariable 1**	Multivariable 2*
Time from baseline (years)				
	<1	1	1	1
	1–2	0·73 (0·67–0·79)	0·72 (0·66–0·79)	0·72 (0·67–0·78)
	2–3	0·58 (0·53–0·64)	0·58 (0·53–0·64)	0·57 (0·52–0·63)
	3–4	0·46 (0·41–0·52)	0·43 (0·38–0·49)	0·44 (0·39–0·50)
	4–5	0·43 (0·38–0·49)	0·41 (0·36–0·47)	0·40 (0·35–0·45)
	5–6	0·38 (0·32–0·44)	0·34 (0·29–0·40)	0·34 (0·29–0·39)
	6–7	0·35 (0·30–0·42)	0·28 (0·23–0·35)	0·30 (0·25–0·36)
	≥7	0·24 (0·21–0·28)	0·18 (0·15–0·21)	0·19 (0·16–0·22)
	p value	p<0·0001	p<0·0001	p<0·0001
Current age (years)				
	<20	1	1	1
	20–25	0·53 (0·37–0·76)	0·47 (0·31–0·70)	0·57 (0·39–0·81)
	25–30	0·45 (0·32–0·63)	0·41 (0·28–0·61)	0·50 (0·36–0·70)
	30–35	0·44 (0·32–0·61)	0·41 (0·28–0·59)	0·50 (0·36–0·70)
	35–40	0·39 (0·28–0·54)	0·39 (0·27–0·57)	0·45 (0·32–0·63)
	40–45	0·39 (0·28–0·54)	0·37 (0·25–0·54)	0·45 (0·32–0·63)
	≥45	0·34 (0·25–0·48)	0·34 (0·23–0·49)	0·41 (0·29–0·57)
	p value	p<0·0001	p<0·0001	p<0·0001
HIV risk group				
	Men who have sex with men	1	1	1
	Black African men	1·88 (1·70–2·05)	1·88 (1·70–2·09)	1·79 (1·63–1·95)
	Black African women	1·60 (1·48–1·72)	1·65 (1·51–1·79)	1·51 (1·39–1·63)
	Other	1·36 (1·25–1·48)	1·36 (1·25–1·49)	1·32 (1·22–1·42)
	p value	p<0·0001	p<0·0001	p<0·0001
Calendar year of ART initiation				
	1998–99	1·32 (1·20–1·46)	2·20 (1·95–2·46)	2·16 (1·95–2·37)
	2000–01	1·06 (0·88–1·16)	1·63 (1·46–1·82)	1·65 (1·49–1·82)
	2002–03	0·97 (0·88–1·06)	1·38 (1·25–1·54)	1·39 (1·27–1·54)
	2004–05	0·94 (0·86–1·03)	1·20 (1·09–1·34)	1·23 (1·12–1·35)
	2006–07	1·00 (0·90–1·09)	1·22 (1·10–1·34)	1·20 (1·09–1·32)
	2008 onwards	1	1	1
	p value	p<0·0001	p<0·0001	p<0·0001
Pre-ART CD4 count (per 100 cells per μL increase)		1·01 (0·99–1·03); p=0·51	1·04 (1·02–1·06); p=0·0008	Not included in model
Pre-ART viral load (per log_10_ increase)		1·00 (0·97–1·04); p=0·86	1·08 (1·04–1·12); p=0·0003	Not included in model

Values are rate ratio (95% CI). p value for categorical variables is global p value (likelihood ratio test). Results from univariable and multivariable Poisson regression models. ART=antiretroviral therapy.

* Model intercept=–1·72.

In addition to time from baseline, older age and more recent calendar year at start of ART were both associated with reduced rates of viral rebound (table 2). Black African men and black African women had significantly higher rates of viral rebound than men who have sex with men. Higher pre-ART viral load and higher pre-ART CD4 count were associated with higher rates of viral rebound in the multivariable model but not the univariable model.

We considered whether predictors of viral rebound were similar for the first 5 years of follow-up and the subsequent follow-up period (data not shown). We found only one significant interaction: being on a regimen other than a non-nucleoside reverse transcriptase inhibitor-based regimen or a ritonavir-boosted protease inhibitor-based regimen at baseline was associated with a reduced risk of viral rebound after 5 years.

When assessing alternative definitions of viral rebound, we observed a decrease over time from baseline in the rate of first viral rebound in all sensitivity analyses (table 3).

**Table 3:** Effect of alternative definitions of viral rebound on the rate of viral rebound

	**Overall**	**From year 7 onwards**
Primary viral rebound endpoint*	7·8 (7·6–8·0)	3·0 (2·6–3·5)
Not including treatment interruption	6·0 (5·8–6·2)	2·3 (1·9–2·7)
Not including treatment interruption but censoring follow-up at the date of treatment interruption	5·3 (5·2–5·5)	2·1 (1·8–2·4)
Including loss to follow-up†	13·7 (13·4–14·0)	8·3 (7·6–9·0)
Two consecutive viral load measures of >200 copies per mL, including treatment interruption	4·9 (4·7–5·1)	2·0 (1·7–2·3)
Not censoring follow-up when a gap in viral load measurement of >12 months occurred	7·8 (7·6–8·0)	3·4 (3·0–3·8)

Data are incidence of viral rebound per 100 person-years (95% CI).

* A single viral load of >200 copies per mL or treatment interruption (for ≥1 month).

† Defined as no viral load measure for ≥12 months or no follow-up after Jan 1, 2013.

We used the estimates derived from the simplified multivariable Poisson regression model (multivariable model 2; table 2) to project the cumulative risk of viral rebound by particular ages, conditional on survival to that age. For an MSM who started ART after 2008 and achieved initial viral load suppression by 9 months from the start of ART at age 35 years, the probability of first viral rebound is 24% (18% if accounting for the fact that 29% of viral rebounds are temporary elevations in viral load) by age 40 years, 48% (38%) by 65 years, and 60% (49%) by 85 years (table 4). For comparison, the probability of viral rebound by age 85 years for people in various example situations are as follows: MSM aged 18 years at the start of ART, 72% (64% if accounting for temporary elevations); black African heterosexual man aged 35 years at the start of ART, 80% (68%); MSM aged 45 years who has been on ART for 8 years without having had first-line viral rebound, 43% (33%). Our estimates suggest that the rate of rebound reaches a plateau of 1·4% per year in MSM older than 45 years, and 1·0% when accounting for the fact that 29% of viral rebounds are temporary elevations.

**Table 4:** Example projections of durability of first-line regimen without viral rebound (assuming ART is started after 2008) in an MSM aged 35 years at different times after ART initiation

		**1 year**	**2 years**	**3 years**	**4 years**	**5 years**	**6 years**	**7 years**	**8 years**	**9 years**	**10 years**	**11 years**	**31 years**	**51 years**
Age (years)		35	36	37	38	39	40	41	42	43	44	45	65	85
Primary viral rebound endpoint														
	Rate of viral rebound (per 100 person-years)	8·1	5·8	4·6	3·6	3·2	2·8	2·5	1·5	1·5	1·5	1·5	1·4	1·4
	Cumulative probability of first-line viral rebound	8%	13%	17%	20%	22%	24%	26%	27%	29%	30%	31%	48%	60%
Primary viral rebound endpoint not including temporary elevations*														
	Rate of viral rebound (per 100 person-years)	5·8	4·1	3·3	2·6	2·3	2·0	1·8	1·1	1·1	1·1	1·0	1·0	1·0
	Cumulative probability of first-line viral rebound	6%	9%	12%	15%	17%	18%	20%	21%	21%	22%	23%	38%	49%

Example of an MSM aged 35 years at 9 months after the start of ART, who has achieved viral suppression of less than 50 copies per mL. Rate is calculated on the basis of parameter estimates from the multivariable model 2 in table 2, including the intercept estimate of −1·72. For example, the viral rebound rate within 1 year from baseline is: exp(−1·72) × 1·00 (rate ratio for <1 year since ART initiation) × 0·45 (rate ratio for age category) × 1·00 (rate ratio for MSM) × 1·00 (rate ratio for ART started in 2008 onwards)=8·1 per 100 person-years. The viral rebound rate at 1–2 years from baseline is: exp(−1·72) × 0·72 (rate for years 1–2) × 0·45 × 1·00 × 1·00=5·8 per 100 person-years. So the probability of not having had viral rebound by 1 year from baseline is exp(−viral rebound rate in year 1)=0·922, and the probability of having had viral rebound is 0·078 (rounded to 8%). The probability of not having had viral rebound by 2 years from baseline is: –exp(−viral rebound rate in year 2) × 0·922=0·870, and the probability of having had viral rebound=0·130 (13%). ART=antiretroviral therapy. exp=exponential. MSM=man who has sex with men.

* Viral rebound after which the next viral load was <50 copies per mL without any change in ART regimen.

## Discussion

In people starting ART who have achieved viral load suppression on a first-line regimen started after 2008, rates of viral rebound are low and decline over 7 years to a low plateau. In MSM older than 45 years, the estimated plateau rate is 1·4% per year. When considering that 29% of viral rebounds consisted of a single measurement of more than 200 copies per mL followed by a subsequent measurement of 50 copies per mL or less, the rate is around 1% per year. These results suggest that many people on ART will not have viral rebound over their lifetime.

We have previously reported (based on data from a shorter follow-up) the decreasing rate of viral rebound with increasing time of viral suppression in people visiting UK clinics.^14^, ^16^ In this Article, we extend that observation with up to 15 years of follow-up and substantially greater numbers of participants. One explanation for the decrease in rate of first viral rebound is that individuals each have their own tendency to adhere to treatment, which is constant over time, so as viral rebounds occur, the remaining population becomes one containing the most adherent individuals. Another is that people's behaviour changes over time and they become accustomed to taking antiretroviral drugs once the drugs are integrated into their everyday lifestyles. A third potential contributing factor is that viral suppression can be maintained with decreasing adherence over time.^19^

In agreement with other studies, we found that older age^15^, ^16^ and later calendar year^13^, ^20^ at start of ART were associated with a reduced risk of viral rebound. Older age is associated with better adherence,^21^, ^22^ which could explain the reduced rate of rebound in older people in our study. Older people might face problems with taking ART because of polypharmacy and incidence of comorbidities, but we found evidence of a lower risk of rebound at older ages, even though there was no trend after age 45 years. The effect of calendar year is likely to be explained by the use of less toxic, more effective drugs in more recent years, which are easier for people to tolerate, but could also be because of improvements in the management of toxic effects and of general health over time. Additionally, we found that being of black African origin is associated with a moderately increased risk of viral rebound, as was found in a protease inhibitor monotherapy trial in the UK.^23^ The reasons for this increased risk are uncertain, although they might relate to socioeconomic conditions or inconsistent prioritisation of ART adherence over other challenges of daily life. We are uncertain about extrapolation of our findings to Africans living in sub-Saharan Africa, because the lower socioeconomic conditions and greater hardships resulting from taking time from work and getting to a clinic in these settings might mean that rebound rates would be higher than in our study.^24^, ^25^, ^26^ However, the proportion of people reported to be on ART and to have viral loads of less than 1000 copies per mL from studies and routine data from sub-Saharan Africa,^12^ most recently from the population-based HIV impact assessments surveys in Malawi (91%), Zimbabwe (87%), and Zambia (89%), suggest that durability of virological suppression could be at least as great in Africa as in the UK.^27^

The possible cause of viral rebound after many years of virological suppression is unknown, and we found no evidence for a limit of durability of ART efficacy. Occasional life events could result in suboptimal adherence that leads to viral rebound. We found that being on regimens other than a non-nucleoside reverse transcriptase inhibitor-based regimen or a ritonavir-boosted protease inhibitor-based regimen at baseline was associated with a reduced risk of viral rebound after 5 years, but we cannot rule out the possibility that this finding might be a type I error.

As in other studies,^28^ we found that a higher viral load at the start of ART was associated with an increased risk of viral rebound in our first multivariable model (although not in our univariate model). After adjustment for viral load at ART initiation in the first multivariable model, we found that a higher CD4 count at the start of ART was also associated with a higher rate of viral rebound; however, we are unsure of the reason for this result. This association is not accounted for by temporary use of ART in pregnant women, for example, because the effect was stronger in men (data not shown).

To our knowledge, this is the largest study to provide long-term estimates of rates of first viral rebound for up to 15 years after ART initiation. Our estimates provide guidance to ART clinics on the expected rates of first viral rebound in people on first-line ART. The results also have implications for the risk associated with condomless sex because of unidentified viral rebound having occurred since the last measure.

The findings presented in this Article should be interpreted in the context of several limitations. Our results are based on people who have not had previous viral rebound and should not be extrapolated to people who do not manage to achieve a good initial virological response to ART in a suitable timeframe or who are receiving second or subsequent lines of ART. Further, we did not collect data on several factors that are likely to be important determinants of adherence, including socioeconomic status, housing stability, drug and alcohol use, mental health diagnoses, and incarceration. We did not collect data on reasons for treatment interruption, and data on interruptions might be incomplete. We cannot rule out that some people who were no longer being followed up in the UK CHIC Study did have viral rebound after they left their participating clinic. Our sensitivity analysis in which people who were no longer being followed up in the UK CHIC Study were defined as having had viral rebound resulted in a 76% higher rate of viral rebound compared with our primary definition. However, many of the participants who fulfilled our definition of having viral rebound did not have true first-line treatment failure (ie, as a result of drug resistance), and could have continued with a first-line regimen even after fulfilling our definition. Another caveat is that we censored follow-up in people who were lost to follow-up or died. Such censoring could have led to bias in our rebound rates because the subsequent rate of viral rebound could be different in those whose follow-up was censored because of loss to follow-up, than in those in whom it was not. We did not consider the assay used to measure viral load, but previous studies have found that the rate of rebound depends on the assay used.^23^

In summary, rates of viral rebound in people with long-term viral suppression on first-line regimens have become extremely low, which suggests that many people on ART will not have viral rebound over their lifetime.
